# Plant Invasions Associated with Change in Root-Zone Microbial Community Structure and Diversity

**DOI:** 10.1371/journal.pone.0141424

**Published:** 2015-10-27

**Authors:** Richard R. Rodrigues, Rosana P. Pineda, Jacob N. Barney, Erik T. Nilsen, John E. Barrett, Mark A. Williams

**Affiliations:** 1 Interdisciplinary Ph.D. Program in Genetics, Bioinformatics, and Computational Biology, Virginia Tech, Blacksburg, Virginia, United States of America; 2 Department of Horticulture, Virginia Tech, Blacksburg, Virginia, United States of America; 3 Department of Plant Pathology, Physiology, and Weed Science, Virginia Tech, Blacksburg, Virginia, United States of America; 4 Department of Biological Sciences, Virginia Tech, Blacksburg, Virginia, United States of America; Shandong University, CHINA

## Abstract

The importance of plant-microbe associations for the invasion of plant species have not been often tested under field conditions. The research sought to determine patterns of change in microbial communities associated with the establishment of invasive plants with different taxonomic and phenetic traits. Three independent locations in Virginia, USA were selected. One site was invaded by a grass (*Microstegium vimineum*), another by a shrub (*Rhamnus davurica*), and the third by a tree (*Ailanthus altissima*). The native vegetation from these sites was used as reference. 16S rRNA and ITS regions were sequenced to study root-zone bacterial and fungal communities, respectively, in invaded and non-invaded samples and analyzed using Quantitative Insights Into Microbial Ecology (QIIME). Though root-zone microbial community structure initially differed across locations, plant invasion shifted communities in similar ways. Indicator species analysis revealed that Operational Taxonomic Units (OTUs) closely related to *Proteobacteria*, *Acidobacteria*, *Actinobacteria*, and *Ascomycota* increased in abundance due to plant invasions. The Hyphomonadaceae family in the Rhodobacterales order and ammonia-oxidizing *Nitrospirae* phylum showed greater relative abundance in the invaded root-zone soils. Hyphomicrobiaceae, another bacterial family within the phyla *Proteobacteria* increased as a result of plant invasion, but the effect associated most strongly with root-zones of *M*. *vimineum* and *R*. *davurica*. Functional analysis using Phylogenetic Investigation of Communities by Reconstruction of Unobserved States (PICRUSt) showed bacteria responsible for nitrogen cycling in soil increased in relative abundance in association with plant invasion. In agreement with phylogenetic and functional analyses, greater turnover of ammonium and nitrate was associated with plant invasion. Overall, bacterial and fungal communities changed congruently across plant invaders, and support the hypothesis that nitrogen cycling bacteria and functions are important factors in plant invasions. Whether the changes in microbial communities are driven by direct plant microbial interactions or a result of plant-driven changes in soil properties remains to be determined.

## Introduction

Invasive plants are implicated in altering plant community dynamics, disturbance regimes, net primary productivity, and nutrient cycles [1–3], which threaten ecosystem functioning and stability. The soil microbial community plays a central role in ecosystem functioning, including serving as plant symbionts, mediating plant nutrient acquisition, nutrient cycles, and soil formation [4]. These belowground communities have been implicated in invasive species success, but only a few studies have assessed how belowground microbial taxa change with plant invasions into ecosystems [5].

Important feedbacks between plants and the soil biotic community have begun to shed new light on plant rarity and invasiveness. High density of native species, such as *Rhododendron maximum*, reduced soil nutrient availability and mycorrhizae abundance associated with surrounding plants [6–9]. *Alliaria petiolata* in contrast, an invasive plant, reduced arbuscular mycorrhizal fungi (AMF) colonization of native trees and overall tree growth [10]. It was thought that the reduction in AMF occurred as a result of the plant releasing glucosinolate containing root exudates [5]. Relatively uncommon native plants were also shown to be more negatively affected by pathogens while invaders, in contrast, showed evidence of more positive plant-microbial feedbacks [11, 12]. These results have been further corroborated using reciprocal transplant studies of plant-soil-microbial feedbacks associated with invaded and native ranges of *Triadica sebifera* [13] and *Pinus contorta* [14]. Still, other effects related to soil nutrient cycling indicated that a mixture of the exotic grasses *Avena barbata* and *Bromus hordeaceous* had elevated levels of nitrate, ammonia oxidizers, microbial N, and gross nitrification rates compared to the native grass *Nasella sp*. [15]. Overall, these results show that microbial communities and their processes are altered due to the invasion of exotic plants, and provide evidence that invader and plants native to an ecosystem have underlying differences in their interactions with belowground microbial communities. Meta-analysis have concluded, specifically, that nitrogen turnover is greatly altered and often greater following exotic plant invasion of ecosystems dominated by native plants [16, 17].

Most of the microbial studies conducted have either been based on greenhouse plantings or field establishment of plants rather than observing changes that occur due to natural invasion in the landscape. There are also few studies that have measured microbial communities in the root-zones of native and invaded soil-ecosystems to determine the structure and composition of microbial communities and whether these field observations corroborate the multitude of different litter-based and experimental observations [18]. A recent meta-analysis suggested the importance of invader-ecosystem interactions and the lack of studies across taxonomic groups and habitats [19]. Meta-analyses help to unify ideas and hypotheses, but can mask the relationship between invasive plant species and their influence on soil nutrient pools and microbial dynamics, which are thought to be quite species specific [20, 21]. Studies that are inclusive of multiple invasive plants and their effects on root-zone microbial community structure and function can thus help to inform whether belowground changes are specific, or broadly associated with plant invasion.

Our overall objective was to understand the effects of plant invasions on soil microbial community structure and its potential linkages to plant-ecosystem function. Specifically, we had two main questions: (1) Do invading species with different taxonomy and phenetic traits have similar or unique effects on microbial communities in root-zone soils?; and (2) are changes in root-zone communities consistent with changes associated with microbial function and soil processes?

## Materials and Methods

### Species and site descriptions

Study sites were selected that met the following criteria: (1) each site must have invaded and non-invaded (reference) areas, the latter of which represents the site pre-invasion; and (2) one invasive species dominates its strata in the invaded plot—no more than 10% cover of other invasive species are located in the invaded plot. Based on these criteria, three sites were selected in the Ridge and Valley Province of the central Appalachian Mountains in Virginia, USA (Table 1). One site (M) was invaded by a C_4_ subcanopy grass (*Microstegium vimineum* [Trin] A. Camus; Japanese Stiltgrass) (Mv), another (R) was invaded by a shrub (*Rhamnus davurica* ssp *davurica* Pall.; Dahurian Buckthorn) (Rd), and the third (A) was invaded by a tree (*Ailanthus altissima* (Mill.) Swingle.; Tree of Heaven) (Aa). All three populations were chosen at locations where a nearby non-invaded reference site was available that was similar in plant community composition, slope, and aspect as the invasion. The native vegetation from these non-invaded sites was used as reference (MvR, AaR, RdR). In all cases it was concluded that the reference site was capable of being invaded, and did not have overarching preexisting difference from the invaded site (Table 1). The term “invasion” is used to differentiate between invaded and non-invaded effects. Two sites were in use for another research grant funded by the USDA Joint Venture program (11-1480-01, 2011–2015). David Carr at the Blandy Experimental Farm provided permission to sample soils in the *Rhamnus* and reference sites. William McShea provided permission to sample soils at the Smithsonian Conservation Biology Institute forest site in *Ailanthus* and reference locations. We obtained permission from Eastern Divide District to sample soils at the Jefferson National Forest site in *Microstegium* and reference locations. The lands were public and no protected species were sampled.

**Table 1:** Details of Sampling Locations.

Location	Invasive Species	Soil Type	Native Species
**A**: Smithsonian Conservation Biology Institute, Front Royal at an elevation of 378m. (Latitude = 38.88553N, Longitude = -78.13844W)	*Ailanthus altissima* (**Aa**)	Montalto loam. Taxonomic class: Fine, mixed, semiactive, mesic Ultic Hapludalfs.	**AaR**: Red oak species (*Quercus species*), tulip poplar (*Liriodendron tulipifera*), and common hackberry (*Celtis occidentalis*). The understory had an abundance of spice bush (*Lindera benzoin*) and infrequent dunal pawpaw (*Asimina triloba*) and bush honeysuckle *(Lonicera maackii*).
**M**: Jefferson National Forest, Montgomery County at an elevation of 2280m.(Latitude = 37.28108N, Longitude = -80.47523W)	*Microste-gium vimineum* (**Mv**)	Berks-Weikert composition on slopes from 15 to 25 percent [22]. Taxonomic class: Loamy-skeletal, mixed, active, mesic Typic Dystrudepts.	**MvR**: The forest canopy is primarily red maple (*Acer rubrum*), white oak (*Quercus alba*), and red oak (*Quercus rubra*). The understory community composition is typical of Appalachian forests of Virginia with total site richness of 78 species [23].
**R**: Blandy Experimental Farm, Boyce at an elevation of 183m. (Latitude = 39.05923N; Longitude = -78.05428W)	*Rhamnus davurica* (**Rd**)	Timberville silt loam. Taxonomic class: Fine, mixed, active, mesic Typic Hapludults Poplimento-Rock outcrop complex. Taxonomic class: Fine, mixed, subactive, mesic Ultic Hapludalfs.	**RdR**: Perennial grasses (e.g., *Panicum virgatum*) and infrequent annual and perennial herbaceous weeds

The following experimental groups were studied: (i) location (A, M, and R); (ii) invasion status (Invasive plants (I) and Native plants (N)); and (iii) interaction of location and invasion status (Aa, AaR, Mv, MvR, Rd, and RdR).

*Microstegium vimineum* is a shade-tolerant C4 annual grass common to much of the Eastern US where it has been implicated in reducing tree recruitment (e.g., [20]), decreasing microarthropod diversity [24], and changing soil chemistry and soil microbial communities [25]. This *M*. *vimineum* invasion is located near an old homestead upslope from the site, but the exact date of establishment is unknown. The reference site was selected across an ephemeral stream likely acting as a barrier to dispersal to the *M*. *vimineum* population.

*Rhamnus davurica ssp*. *Davurica* is a deciduous short-lived shrub native to China, North Korea, Mongolia, eastern Siberia and Japan. It was commonly planted in the Northwestern US plains for windbreaks in the 1930’s. Both *R*. *davurica* and *Rhamnus cathartica* L. (Common Buckthorn) were incorporated into the Virginia Arboretum in 1939, but only *R*. *davurica* has invaded into the Blandy Experimental Farm in Boyce, Virginia, USA. The site invaded by *R*. *davurica* has been unmanaged for over 3 decades and has not for the Blandy Experimental Farm. The *R*. *davurica* invasion into the grassland is well documented at this farm and has occurred over a 25-year period.

*Ailanthus altissima* is a common urban, roadside, and natural area invasive tree capable of growing in a variety of non-managed and disturbed systems worldwide; spreading both sexually and clonally [20, 25, 26]. This fast growing tree has putative allelopathic effects [23], though the ecological impacts of *A*. *altissima* are largely unknown [27]. The *A*. *altissima* invasion occurred at this site over the last 40 years following a clear cut on one side of a logging road. The other side of the road was not logged and is an non-invaded reference area. While logging removed overstory vegetation, the impacts on soil were relatively small.

### Soil sampling and analyses

Soil sampling locations were selected by a stratified random technique. A 50 m transect was established along one edge of each plot (same for both invaded and non-invaded plots). The transect was divided into five replicate 10 m reaches. A random number generator was used to pick a meter mark within each 10 m reach for establishing a perpendicular transect. Once the position of the transect was identified, the random number generator was used to select a distance along the perpendicular transect for the soil sample. At this location, a coin was flipped to choose the right or left side of the perpendicular transect to sample. The soil sample was taken 1 m away from the perpendicular transect. If the final location was occupied by a rock or tree, the closest location where a soil sample could be taken was used. Soils were sampled at each location using a standard 7-cm soil corer (Model # 402.25, AMS Inc., American Falls, ID, USA).

At each sample location, the litter and humus layers were removed. The soil corer was washed with 95% alcohol before sampling and between each soil sample. The soil sampler was then driven in to a depth of 10 cm using a professional slide hammer (Model 57780, AMS Inc., American Falls, ID, USA). Leaf litter, roots, and large debris were removed from each sample (100 cm^3^) and the soil samples were placed in a sterile zip-top bag and refrigerated in a cooler until the samples could be stored at -5°C in the lab at Virginia Tech. This resulted in ten randomly selected soil samples at each site, five of which were from the invaded and five from the adjacent non-invaded reference. Each soil sample was sieved through an alcohol washed #20 soil sieve (Model H-3903, M & L Testing equipment, Calgary, Alberta, Canada), and individually mixed and homogenized. All precautions against contamination were taken. Subsamples of the sieved soil were analyzed for several nutrient cations and anions, extractable nitrogen, and microbial diversity. The subsamples for nutrient cation analysis were extracted with 1*M* KCL and analyzed using ICP. Soil parameters measured were: pH, cation exchange capacity, and concentrations of P, K, Ca, Mg, Zn, Mg, Cu, Fe, and B.

A separate subsample was incubated for seven days at field moisture water potential. Directly before and following the seven days of incubation, samples were extracted with 1M KCl to determine extractable inorganic nitrogen content. Based on water content and particle size analysis, it was estimated that water potential for all soil samples ranged between -100 to -500 KPa. Sampling in May ensured that each sample was near saturation and similarly moist. Total nitrate and ammonium ions (μg g^-1^) were measured with a Lachet autoanalyzer (Quikchem 8500 Series 2) and turnover (T_1_-T_0_) x (100/ T_0_) was calculated following a one week incubation of soil (25°C). Wilcoxon (rank-sums) test with a normal approximation to the two-sample test was performed in JMP^®^ Pro, Version 11 (SAS Institute Inc., Cary, NC, 1989–2007) to check whether the turnover was different between invaded and non-invaded samples. Microbial community structure and diversity were determined on another subsample of soil DNA (see below).

### Univariate statistical analysis on soil nutrients

A two-way analysis of variance was used to determine significant effects of location, invasion status (invaded or non-invaded), and their interaction on soil nutrition. Means were separated using Tukey HSD at alpha = 0.05. All ANOVAs were performed with JMP statistical software (SAS Institute Inc., Cary, North Carolina).

### DNA extraction and amplification

For both the 16S rRNA gene analyses and the ITS analyses, 0.5 g of freeze-dried homogenized soil was weighed and DNA was extracted from each soil sample using PowerSoil^®^ DNA Isolation Kit (MoBio) according to the manufacture’s protocol. DNA quality was checked on a 0.8% (w/v) agarose gel. DNA concentrations were determined by fluorometric quantification using the Qubit^®^ 2.0 platform with Qubit dsDNA HS Assay Kit (Life Technologies). DNA was diluted to 50 ng μL^-1^ and stored in a -20°C freezer. It was used for the PCR-based protocol described in [28], using the PCR bacteria/archaeal primers 515F/806R targeting the V4 region of the 16S rRNA. ITS1FI2/ ITS2R were used to amplify the spacer ITS1 of the internal transcribed spacer (ITS) rDNA region [29, 30]. The reverse amplification primer also contained a twelve base barcode sequence. Both PCR primers contain sequencer adapter regions. The enzyme used in the PCR reaction was KAPA2G Robust (5 U/μL) from Kapa Biosystem. For 16S rRNA assay the 25 μL reaction mixture contained 0.5 μL of dNTPs (10 mM), 0.5 μL of each primer (10 μM), 50 ng of the DNA template, 1 μL of DMSO (100%), 0.2 μL of the enzyme (5U/μL) and 5 μL of Buffer GC (Kapa Biosystem). For the ITS assay, the PCR reaction final volume was 25 μL, containing 0.5 μL of dNTPs (10 mM), 0.625 μL of each primer (10uM), 50 ng of the DNA template, 1.25 μL of DMSO (100%), 0.2 μL of the enzyme (5 U/μL) and 5 μL of Buffer A (Kapa Biosystem). The PCR conditions used were as follows: for the 16S assay, there was a denaturation step at 94°C for 3 minutes, 35 cycles of 94°C for 45 seconds, an annealing step at 60°C for 60 seconds, an extension step at 72°C for 90 seconds, and a final extension at 72°C for 10 minutes. For the ITS assay, there was a denaturation step at 95°C for 15 seconds, 35 cycles of 95°C for 30 seconds, an annealing step at 55°C for 30 seconds, an extension step at 72°C for 30 seconds, and a final extension at 72°C for 5 minutes. The specificity of the PCR was further evaluated by running on a 1.2% (w/v) agarose gel. The concentration of the DNA was obtained by Fluorometric Quantitation (Qubit^®^ 2.0 Life Technologies) before sending samples to sequencing. From the bacterial experiments, two out of the 30 samples did not show 16S rRNA gene amplification. Hence, 28 samples were sent for 16S rRNA gene sequencing, whereas all 30 samples were sent for ITS rDNA sequencing. Sequencing on the Illumina MiSeq platform was conducted by the Virginia Bioinformatics Institute core facility.

### Sequence data analyses

In the bacterial data, an ‘Rd’ sample (F8) was removed from further analysis due to contamination on the sequencing plate. The paired end reads were stitched using *Pandaseq* [31]. For the fungal data, only read-2s with a quality threshold of 30 were used for further analyses. The bacterial and fungal sequencing data were analyzed using *QIIME* [32]. Briefly, reads were clustered into OTUs based on 97% sequence similarity using *uclust* [33] and *usearch61* [33], for bacteria and fungi respectively, using an open reference OTU-picking strategy. The representative sequence of an OTU was used to assign it a taxonomy, using *uclust* against the Greengenes reference database version 13_8 [34, 35] for bacteria, and *RDP classifier* [36] against the UNITE reference database version 12_11 [37] for fungi.

### Comparison and statistics on groups

A sampling depth threshold of 80,000 and 3,200 sequences per sample, for bacteria and fungi respectively, was used for the diversity and taxonomic summary analyses. The beta diversity was calculated using weighted and unweighted Unifrac [38] (for bacteria), and Bray-Curtis [39](for fungi) distance metrics. To identify group differences, the distances were used for Principle Coordinate Analysis [40] and visualized in 3D-plots using EMPeror [41]. The chao1 [42] and observed species metrics were used to plot alpha rarefaction curves. The alpha diversity was calculated using PD whole tree (for bacteria only), chao1, observed species, and Shannon and Simpson indices for bacteria and fungi. The bar graphs with standard error bars were used to visualize microbial taxonomic summaries of the interaction between location and invasion at different levels and generated using custom python scripts. Multivariate data analysis methods of adonis [43] and Analysis of Similarity (ANOSIM) [44] were used to identify whether groups were significantly different. Indicator species analysis (ISA) [45] in PC-ORD Version 6 [46] was used to identify taxa that were significantly (indicator value > 70 and p-value < 0.01) associated with invasion when blocked by geographic sites/location. A seed of 16 and 18 with 5000 runs was used for the bacteria and fungi, respectively.

### Functional analyses

The actual abundance (counts) of the OTUs belonging to the significant genera from ISA was used for functional analyses using *PICRUSt* [47]. OTUs not part of the closed reference OTU picking were filtered out. Using default parameters, the filtered OTU table was normalized by the 16S rRNA copy number abundance to identify true abundance followed by metagenome functional prediction for each sample. The metagenomes were collapsed into KEGG pathways. Using STAMP [48], two-sided Welch's t-test [49] with Benjamini-Hochberg [50] and Storey [51] multiple testing corrections were performed to identify KEGG pathways that were significantly different (q-value < 0.05) between invaded and non-invaded samples.

## Results

### Soil nutrients change associated with invasion

Many soil parameters, particularly pH, P, K, Mg, Zn, and B varied among locations (Table 2). Four soil parameters varied between invaded and non-invaded plots across locations (Table 2). Interestingly, 7 of the 11 soil parameters varied between invaded and non-invaded plots among species, including pH, P, and CEC (Table 2).

**Table 2:** Mean Values (St. Dev.) and Two-Way Analysis of Variance on Soil Nutrition Parameters from Three Sites in Central Appalachian Mountains with Invaded and Non-Invaded Locations.

Location	M		R		A		Location	Invasion Status	Location x invasion status
Invader	*Microstegium vimineum*		*Rhamnus davurica*		*Ailanthus altissima*				
Invasion status	Invaded	Non-invaded	Invaded	Non-invaded	Invaded	Non-invaded			
pH	5.36** (0.27)	4.9 (0.15)	6.69* (0.2)	6.66 (0.12)	6.29* (0.12)	6.67 (0.31)	**<0.001**	0.608	**0.001**
P	2.4 (0.5)	2.2 (0.5)	11.8 (6.8)	4.4 (1.1)	2.6 (0.8)	2.0 (0.00)	**<0.001**	**0.015**	**0.016**
K	106.8** (28.3)	52.4 (5.9)	104.0* (23.8)	72.4 (18.5)	150.4 (35.8)	126.4 (37.6)	**0.002**	**0.002**	0.442
Ca	553.2** (208.1)	156.2 (26.3)	1151.6 (175.9)	1123.6 (117.4)	1174.0* (285.4)	1634.8 (265.6)	**<0.001**	0.872	**0.000**
Mg	65.0 (11.2)	32.8 (2.6)	97.0 (7.3)	88.4 (9.2)	164.0 (46.8)	208.4 (25.7)	**<0.001**	0.887	**0.003**
Zn	2.18 (0.37)	1.88 (0.29)	1.36 (0.31)	1.28 (0.25)	4.72 (1.18)	5.12 (1.11)	**<0.001**	0.980	0.538
Mn	12.62*** (1.12)	15.4 (7.61)	11.44*** (5.7)	7.48 (0.64)	14.96** (2.58)	32.3 (7.74)	0.478	**0.004**	**<0.001**
Cu	1.4 (0.22)	1.48 (0.50)	0.60 (0.23)	0.82 (0.18)	1.24 (0.55)	0.78 (0.19)	**0.003**	0.677	0.091
Fe	18.5 (4.93)	22.4 (5.37)	16.4 (18.1)	18.5 (4.93)	3.98* (1.08)	2.46 (0.67)	**0.027**	0.118	0.185
B	0.3** (0.1)	0.2 (0.0)	0.5 (0.1)	0.5 (0.1)	0.8** (0.2)	1.4 (0.3)	**0.000**	**0.017**	**<0.001**
CEC	6.4 (0.42)	6.1 (1.16)	6.9 (0.86)	6.5 (0.62)	8.14 (1.60)	10.26 (1.50)	0.052	0.222	**0.034**

Parameter = soil nutrition trait; Location = the three locations where each species was sampled; Invasion Status = invaded and non-invaded plots. Bolded values indicate significant (p ≤ 0.05) effects. All nutrient units are μg element g^-1^ soil. The statistical test (Tukey HSD means separation) is between invaded and non-invaded within site.

* = p ≤ 0.05

** = p ≤ 0.01

*** = p ≤ 0.001

In most cases, nutrient parameters were higher in the invaded patch compared to the non-invaded patch (Table 2). For example, *Microstegium vimineum* increased pH, K, and Ca, *Rhamnus davurica* increased K and Mn, while *Ailanthus altissima* lowered pH, Ca, Mn, Fe, and B (Table 2).

Concentrations of nitrate in soil ranged from 1.5 to 18.3 and ammonium from 9 to 29 μg g^-1^ soil. Following one week of incubation (22°C), the concentrations increased, on average, ranging from non-detectable to 24 for nitrate and 33 to 51 μg g^-1^ ammonium. Wilcoxon (rank-sums) test with a normal approximation to the two-sample test showed that turnover of nitrate during the one week incubation was observed to be significantly greater in association with invasion (p-value = 0.014), averaging 137 and 61 percent per week of incubation in invasive and non-invasive factors, respectively (Table 3). On the other hand, turnover of ammonium during the one week incubation was observed to be greater, but not significant, in association with invasion, averaging 154 and 123 percent per week of incubation in invasive and non-invasive factors, respectively. These results suggest that invasion increased the rate of N cycling and availability of nitrogen for plant uptake from soil. The results also agree with the phylogenetic and functional analyses which showed greater N cycling genes, and greater relative abundance of nitrifying and putative nitrogen-fixing bacteria in the invasive compared to non-invasive soil.

**Table 3:** Turnover (Percentage) of Inorganic Nitrogen (Mean, SE^a^) in Non-Invaded and Invaded Locations at Three Sites in Central Appalachian Mountains.

Location	M		R		A			
Invader	*Microstegium vimineum*		*Rhamnus davurica*		*Ailanthus altissima*		*All plant species*	
Invasion status	Invaded	Non-invaded	Invaded	Non-invaded	Invaded	Non-invaded	Invaded	Non-invaded
NO_3_	42 (8)	-20 (20)	236 (106)	196 (70)	108 (24)	33 (5)	137 (45)	61 (31)
NH_4_	247 (41)	347 (45)	6 (19)	-61 (20)	209 (48)	83 (17)	154 (35)	123 (48)

^a^ The standard error (SE) of the mean is in given in parenthesis.

### Alpha diversity of microbial communities associated with invasion

#### Bacteria

A total of ~17.8 million high quality 16S rRNA gene sequence reads were obtained from the invaded and non-invaded plots. The sequences from 27 samples possessed a 254-bp average length and will be submitted to the NCBI Sequence Read Archive according to MIMS standard. There were a total of 210,007 distinct OTUs (observations) across samples with a total of 4,444,765 sequences (counts) that were assigned to these OTUs. The observation refers to the number of distinct OTUs; whereas the count refers to the abundance of bacteria belonging to these OTUs in samples. The mean and median counts per sample were 164,621 and 158,958, respectively. A sampling depth threshold of 80,000 counts per sample removed one sample from further analyses. The average Good’s coverage for the bacterial data across 26 samples was 96.1%.

Chao1, observed species, Shannon, Simpson, and PD whole tree metrics were used to calculate alpha diversity (species diversity within the community). A non-parametric test with the default 999 Monte Carlo permutations with an FDR correction showed significant differences (α<0.05) between locations and between location x invasion for alpha diversity but not between invaded and non-invaded samples (Shannon and Simpson metrics were not used) (Data not shown). However, the rarefaction curves, which are sample size independent, showed trends that non-invaded samples have lower alpha diversity (S1 Fig). Without the sampling depth threshold on the 26 samples, a one-tail Mann-Whitney test showed that the alpha diversity of invasive samples was significantly greater (α<0.05) than that in non-invaded samples for all five diversity metrics (Table 4). Kruskal Wallis test with a Chi-Square approximation of one-way test in JMP^®^ Pro, Version 11 (SAS Institute Inc., Cary, NC, 1989–2007) suggested that the diversity metrics (except Simpson index) were significantly different (α<0.05) between samples as per location and interaction of location and invasion status. Since the sample size variation can affect the diversity metrics, the sampling depth threshold was utilized for further analyses by taking a random subsample of 80,000.

**Table 4:** Alpha Diversity Metrics for Invasion, Location, and Location x Invasion in Bacteria.

	Chao1	Observed Species	Shannon	Simpson	PD Whole Tree
Invasion status					
I (n = 11)	24,563	15,024	10.83	0.998	604
N (n = 15)	20,566	12,328	10.54	0.997	512
**p-value (one-tail)**	**0.012**	**0.004**	**0.006**	**0.007**	**0.007**
Locations					
A	25,687	15,326	10.82	0.997	616
M	17,512	11,000	10.43	0.998	448
R	23,460	13,987	10.75	0.997	591
**p-value (two-tail)**	**0.001**	**0.002**	**0.003**	0.817	**0.002**
Location x Invasion status					
Aa	27,684	16,806	10.98	0.998	666
AaR	23,691	13,845	10.65	0.997	566
Mv	19,507	12,108	10.61	0.998	494
MvR	15,915	10,114	10.28	0.997	410
Rd	26,875	16,398	10.87	0.998	668
RdR	22,093	13,023	10.70	0.997	559
**p-value (two-tail)**	**0.003**	**0.002**	**0.002**	0.103	**0.002**

Bolded values indicate significant (α<0.05) effects.

#### Fungi

The read 1s were not used for the analysis due to the poor quality of sequences. A total of 204,835 high quality read 2s of the ITS gene sequence were obtained from the invaded and non-invaded plots. The sequences from 30 samples possessed a 230-bp average length and will be submitted to the NCBI Sequence Read Archive according to the MIMS standard. There were a total of 4,419 distinct OTUs (observations) across samples with a total of 182,009 sequences (counts) that were assigned to these OTUs. The mean and median counts per sample were 6,067 and 4,927 respectively. A sampling depth threshold of 3,200 counts per sample did not remove any sample from further analyses. The average Good’s coverage for the fungal data across 30 samples was 95.5%.

Chao1, observed species, Shannon, and Simpson metrics were used to calculate alpha diversity. A non-parametric test with the default 999 Monte Carlo permutations with FDR correction showed significant differences (α<0.05) between locations, invasion status, and their interaction (location x invasion status) for alpha diversity (Shannon and Simpson metrics were not used) (Data not shown). Similarly to the bacterial data, the rarefaction curves showed trends that non-invaded samples have lower alpha diversity (S2 Fig). Without the sampling depth threshold, a one-tail Mann-Whitney test showed that the alpha diversity of invasive samples is significantly higher (α<0.05) than that in non-invaded samples for chao1 and observed species metrics (Table 5). Kruskal Wallis test with a Chi-Square approximation of one-way test in JMP^®^ Pro, Version 11 (SAS Institute Inc., Cary, NC, 1989–2007) suggested that the diversity metrics were significantly different (α<0.05) between samples as per locations and interaction of locations and invasion status. Since the sample size variation can affect the diversity metrics, the sampling depth threshold was utilized for further analyses by taking a random subsample of 3,200.

**Table 5:** Alpha Diversity Metrics for Invasion, Location, and Location x Invasion in Fungi.

	Chao1	Observed species	Shannon	Simpson
Invasion status				
I (n = 15)	814	537	6.30	0.947
N (n = 15)	728	483	5.87	0.935
**p-value (one-tail)**	**0.039**	**0.023**	0.076	0.221
Locations				
A	863	600	6.60	0.962
M	800	512	6.29	0.959
R	650	420	5.36	0.902
**p-value (two-tail)**	**0.015**	**0.022**	**0.006**	**0.006**
Location x invasion status				
Aa	844	570	6.65	0.965
AaR	883	629	6.55	0.959
Mv	935	604	6.56	0.961
MvR	666	421	6.02	0.958
Rd	664	438	5.68	0.916
RdR	636	401	5.03	0.889
**p-value (two-tail)**	**0.011**	**0.014**	**0.020**	**0.030**

Bolded values indicate significant (α<0.05) effects.

### Beta diversity of microbial communities associated with invasion

#### Bacteria

Multivariate data analyses using adonis, ANOSIM, and MRPP on weighted and unweighted Unifrac distances showed significant differences (α<0.01) in the beta diversity of the location and the interaction of location and invasion status.

#### Fungi

The beta diversity of location, invasion status, and their interaction were significantly different (α<0.01) as shown by adonis, ANOSIM, and MRPP on Bray-Curtis distances, with an exception of ANOSIM indicating a p-value of 0.014 for invasion.

**Fig 1:**
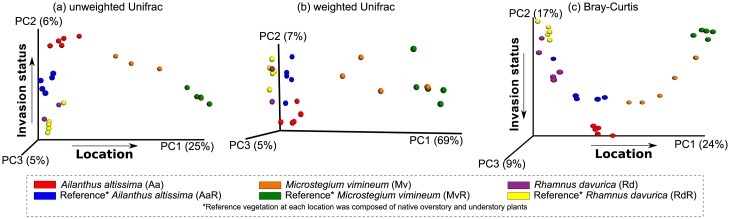
PCoA plot describing (a) un-weighted and (b) weighted Unifrac for bacteria and (c) Bray-Curtis distances for fungi in the invaded and non-invaded sites. Each circle indicates a sample. Multivariate data analysis methods of adonis and ANOSIM were used to identify whether groups were significantly different.

The PCoA analysis of the weighted and unweighted Unifrac (for bacteria), and Bray-Curtis (for fungi) distances showed that the samples clustered as per the location and invasion (Fig 1), with location explaining the maximum variation (PC1). For the unweighted Unifrac and Bray-Curtis distances, invasion status (across all locations) consistently accounted for the second most variation (6% for bacteria and 17% for fungi on PC2). There was a lot of variation associated with the Rd samples as shown in Axis 2 of Fig 1. Overall, these results indicated the effects of invasion and location x invasion status. There were, thus clear patterns of change in soil microbial communities following the invasion of each species across geographically separated ecosystems.

#### Taxonomic summary and identification of microbial communities associated with invasion

**Fig 2:**
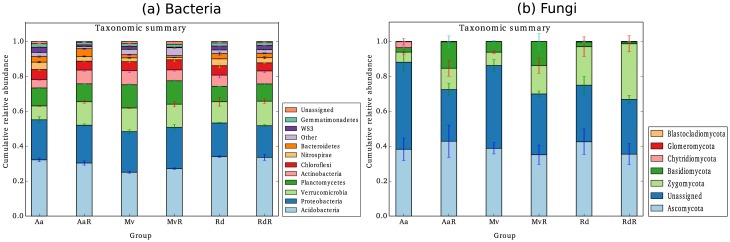
Taxonomic summary of the relative abundance of (a) bacterial and (b) fungal phyla in the invaded and non-invaded sites. The taxa are arranged as per total relative abundance across all samples, with the most abundant phyla at the bottom and the least abundant phyla at the top of the y-axis. Similarly, the phylum names in the legend are arranged from the least abundant at the top to the most abundant at the bottom.

Taxonomic summaries showed that *Acidobacteria* (~30%) and *Proteobacteria* (~22%), and *Ascomycot*a (~47%) and *Zygomycota* (~13%) were the most dominant phyla of bacteria and fungi, respectively (Fig 2). A major proportion of taxa could not be assigned (~34%) to known taxa for the fungal data; however, they were a very minor portion for bacteria. The genus level taxonomic summaries were used for indicator species analysis (ISA) to identify taxa that were more abundantly associated with invaded or non-invaded samples (Table 6). Overall, the results suggested numerous types of taxa associated with invasion, whereas only one taxa was associated with non-invasion.

**Table 6:** Genera with a Greater Relative Abundance Associated with Invasion and Determined to have a Significant Effect Based on Indicator Species Analysis (IV > 70 and p-value < 0.01).

**Bacteria**						
**Phylum**	**Class**	**Order**	**Family**	**Genus**	**I (%)**	**N (%)**
Acidobacteria	-	-	-	-	0.30	0.17
Acidobacteria	Holophagae	Holophagales	Holophagaceae	Geothrix	0.01	0.00
Acidobacteria	iii1-8	SJA-36	-	-	0.03	0.01
Acidobacteria	RB25	-	-	-	0.25	0.12
Acidobacteria	S035	-	-	-	0.08	0.05
Actinobacteria	Actinobacteria	Actinomycetales	Micrococcaceae	Arthrobacter	0.02	0.01
Actinobacteria	Actinobacteria	Actinomycetales	Williamsiaceae	Williamsia	0.01	0.00
Chloroflexi	TK10	-	-	-	0.02	0.01
Gemmatimonadetes	Gemmatimonadetes	-	-	-	0.03	0.01
Nitrospirae	Nitrospira	Nitrospirales	-	-	0.02	0.00
Nitrospirae	Nitrospira	Nitrospirales	Nitrospiraceae	Nitrospira	0.52	0.10
OD1	SM2F11	-	-	-	0.01	0.00
OP3	koll11	-	-	-	0.01	0.00
OP3	PBS-25	-	-	-	0.01	0.00
Proteobacteria	Alphaproteobacteria	Rhodobacterales	Hyphomonadaceae	-	0.21	0.07
Proteobacteria	Alphaproteobacteria	Rhizobiales	Hyphomicrobiaceae	Hyphomicrobium	0.06	0.02
Proteobacteria	Betaproteobacteria	Methylophilales	Methylophilaceae	-	0.01	0.00
Proteobacteria	Betaproteobacteria	Rhodocyclales	Rhodocyclaceae	Dechloromonas	0.03	0.00
Proteobacteria	Deltaproteobacteria	NB1-j	MND4	-	0.17	0.05
Proteobacteria	Deltaproteobacteria	Desulfuromonadales	Geobacteraceae	Geobacter	0.05	0.02
WS2	SHA-109	-	-	-	0.06	0.03
*Actinobacteria	Actinobacteria	Actinomycetales	Thermomonosporaceae	Actinomadura	0.00	0.01
**Fungi**						
**Phylum**	**Class**	**Order**	**Family**	**Genus**	**I (%)**	**N (%)**
Ascomycota	-	-	-	-	1.34	0.34
Ascomycota	Dothideomycetes	Capnodiales	Mycosphaerellaceae	Cladosporium	0.05	0.01
Ascomycota	Leotiomycetes	-	-	-	0.37	0.11
Ascomycota	Sordariomycetes	Hypocreales	Nectriaceae	-	6.52	2.10
Ascomycota	Sordariomycetes	Hypocreales	Nectriaceae	Cylindrocarpon	0.95	0.45
Ascomycota	Sordariomycetes	Hypocreales	Nectriaceae	Fusarium	0.83	0.14
Ascomycota	Sordariomycetes	Hypocreales	Nectriaceae	Neonectria	0.15	0.02
Ascomycota	Sordariomycetes	Incertae sedis	Plectosphaerellaceae	Plectosphaerella	0.24	0.03
Ascomycota	Sordariomycetes	Sordariales	-	-	1.07	0.53

The hyphen (-) indicates that no taxonomic information was available for that OTU at that level. The bacterial OTU indicated with asterisk (*) was the only OTU associated with non-invaded samples in the ISA. The last two columns indicate the percentage of relative abundance of taxa in the invaded and non-invaded samples, respectively.

#### Bacteria

After removing OTUs assigned to archeal and unassigned taxa, OTUs with a total relative abundance of less than 0.1% across all samples were removed. The remaining 416 taxa were re-relativized and used for ISA blocked using soil/geographic locations. Out of 22 OTUs (Table 6) that showed significantly different abundance in invaded and non-invaded samples, 21 OTUs were associated with invasion. OTUs within *Proteobacteria* (6 OTUs), *Acidobacteria* (5 OTUs), and *Actinobacteria* (3 OTUs) had greater sequence abundance due to invasion as revealed by ISA and blocked across soil/geographic locations. Bacterial taxa responsible for nitrogen cycling in soil were increased in abundance in association with plant invasion. Taxa belonging to the ammonia-oxidizer *Nitrospirae* (phylum) and Nitrospira (class) were among the bacteria each with 1.5 times greater abundance in the invaded (3.5% compared to 2.4% in non-invaded) root-zone soils. Nitrifying bacteria appear to be a major result and perhaps driver of invasive plant species change in ecosystems.

The nitrogen-fixing bacterial community was also an important potential indicator of change noted in plant invasions. Several bacterial groups which are known to contain taxa involved in nitrogen-fixation were shown to increase as a result of plant invasion in our data. Rhodobacterales are commonly identified as nitrogen-fixing bacteria [52], and found to collectively contribute to (2.7 times) greater abundance in the invaded root-zone soils in our data (0.22% compared to 0.08% in non-invaded) and previous literature [53]. Hyphomicrobiaceae, another bacterial family within the phyla *Proteobacteria* were also greater as a result of plant invasion, but the effect was most strongly associated with the root-zones of *M*. *vimineum* (1.4 times abundant, 3.7% compared to 2.6% in non-invaded) and *R*. *davurica* (1.2 times abundant, 1.7% compared to 1.4% in non-invaded). Though nitrogen-fixation symbiosis are not widely considered key traits among the invasive plant species in this research study, the greater relative abundance of these putative diazotrophic taxa support the idea that these traits may be important associations for many plant invader types.

#### Fungi

After removing OTUs assigned to unassigned taxa, OTUs with a total relative abundance of less than 0.1% across all samples were removed. The remaining 226 taxa were re-relativized and used for ISA blocked across soil/geographic locations. All of the 9 OTUs (Table 6) that showed significantly different abundance in invaded and non-invaded samples were associated with invasive samples. OTUs within Ascomycota (9 OTUs) had a greater sequence abundance due to invasion as revealed by ISA blocked across soil/geographic locations. Taxa belonging to the Sordariomycetes were among the fungi with 1.2 times greater abundance in the invaded (21.3% compared to 17.5% in non-invaded) root-zone soils.

### Predicting microbial functions in non-invaded and invasive samples

Currently, PICRUSt can only be used for functional analysis of bacterial taxa. To the best of our knowledge, we could not find a program for functional analysis of fungi, analogous to PICRUSt for bacteria. The fungal data resources AFTOL (http://aftol.org/) and FunSecKB [54] provide relevant but incomplete data for our purpose.

The actual counts from the OTU table were obtained for the bacterial species belonging to the genera that were significant from the ISA. OTUs not part of the closed reference OTU picking method were filtered out from the 3,385 OTUs belonging to the 22 significant genera and the remaining 365 OTUs (~11%) were used for functional analyses using PICRUSt. The 16S rRNA copy number normalized abundance was used to predict metagenome and collapse into KEGG pathways. Two-sided Welch’s t-test with multiple testing corrections in STAMP was performed to identify KEGG pathways at different levels that are significantly different (q-value < 0.05) between invaded and non-invaded samples. At Level 2 of KEGG, BH and Storey corrections found 9 and 27 pathways, respectively, to be significantly different between root-zone bacteria of invaded and non-invaded samples (S1 and S2 Tables). At Level 3 of KEGG, BH correction did not detect pathways to be significantly different between invaded and non-invaded root-zone bacteria. However, for the same level, Storey FDR detected 60 pathways to be different (S3 Fig). The significant processes were descending sorted as per the average of mean relative frequency (%) in non-invaded and invaded samples. The top 20 abundant processes were categorized as belonging to non-invaded (N) or invaded (I) samples depending on the difference of mean relative frequency (%) (S3 Table).

As expected from the taxonomies of bacteria from the ISA, nitrogen metabolism was also observed to be higher in the root-zone bacterial communities of invasive plants as compared to that of the non-invaded plants (S3 Table). The increase in nitrogen metabolism by invasive plants and the associated benefits to invasion are well known [55–57].

## Discussion

Plant invasion theory has developed a broad number of hypotheses to explain the success of invasive plants [58]. Despite their likely importance, however, there is a dearth of research into aboveground-belowground linkages across landscape scales that have determined the effects of plant invasion on soil or root-zone microbial communities [59, 60]. Here we show that at three independent locations, three invasive plants are associated with uniform shifts in belowground root-zone soil microbial communities. This is important, further, because each of the invasive plants has a distinct phylogeny and life form. Our results are broadly relevant because belowground interactions between soil microbes and plants provide an important linkage to support plant invasions.

### Bacterial community shifts due to plant invasion

Compared to adjacent non-invaded patches, fungal and bacterial communities were described by consistent ordinal shifts associated with invasion. Nitrospira sp. and *Nitrospirae* were among the bacteria with greater abundance in the invaded soils. Overall *Nitrospirae* was very abundant, and greater in the invaded (3.5% compared to 2.4% in non-invaded) root-zone soils. Previous studies have shown that *Nitrospirae*, which are most often found to be chemolithic autotrophs, and include taxa that are drivers of nitrification, tend to account for 0.2 to 0.7% of OTUs in grasslands, agricultural systems, and forests [61, 62]. However, 2% or more have been observed in remnant deciduous forests [63], which is consistent with the forests described herein. Furthermore, our results corroborate that plant invasions are associated with major changes in the nitrogen cycle [18, 60] by showing greater rates of root-zone soil N turnover due to invasion.

Importantly, the results of our experiments support a major mechanism of plant invasion success, and link microbial phylogeny with functional measurements of nitrogen turnover. The greater rates of nitrogen turnover and estimates of metagenome composition and function using PICRUSt are in agreement that N cycling processes are important components of invader success. Nitrogen-fixing bacterial communities are also an important indicator of change previously documented in plant invasions [64]. The link between nitrogen-fixation and bacterial phylogeny, however, is not as strong as that with nitrification. Several bacterial groups which are known to contain taxa well known for nitrogen-fixation were observed to increase in our study as a result of plant invasion. Nitrogen-fixers can be free-living, and their abundance in soil tends to be low (2.4x10^5^ copies g^-1^); however, associative diazotrophs are generally more common (1.3x10^7^ copies g^-1^) in the root-zones of numerous types of plants if carbon is available to drive the energetically expensive process of N_2_ reduction to ammonium [65]. Since these bacteria are closely linked to plant roots, their greater abundance, and the confirmation that nitrogen fixation genomes are available to support greater nitrogen fixation (PICRUSt) associated with invaded soils, are in support of the argument that the result is not due to *a priori* soil habitat differences, but rather the impact of the root-zones of plant invaders. If greater N-fixation is the result of increasing abundance of diazotrophs, then greater supplies of N could help to foster greater nitrogen availability for plants and nitrifiers alike. These types of interactions have the potential to act as a positive feedback to support the habitat needs of the invader. Negative consequences of increased nitrogen-fixation and nitrification could also come from the leaching of nitrate to groundwater and gaseous losses through denitrification (N_2_O).

Connections between plant traits and root-zone associated microbial communities have been considered [19, 60]. Less work, however, has been conducted to determine how root-zone soil microbes directly benefit and support the longer-term spread of invasive plants [66]. Though the work presented here does not directly address the long-term nature of invasion, they are representative of fairly mature invasions (>5y) and the potential consequences of changing microbial communities and alterations in ecosystem nutrient cycles.

The field results presented help to fill a major gap in understanding plant invaders and mechanisms of invasion success. The evidence provided in the research reported here are consistent with the idea that plant invaders shape belowground communities, and positively feedback to support the success of the plant invader. In addition, the research has shown that plant invaders are associated with change in soil properties which might be driven by the plant invader and facilitated by positive feedbacks resulting from microbial community processes. Alterations in nutrient cycling have previously been described as potential drivers that feedback to support plant invasion. Often these results are tied to changes in plant tissue chemistry and the decomposition [21, 25] but less attention has been paid to the potential effects that plant roots might have more directly on soil nutrient bioavailability. Plant root systems have the capacity to alter soil pH and therefore chemical equilibria and pH sensitive biological processes. Nitrification, for example, has been described as limited by pH below 5.5–6.0 [67]. Chemical equilibrium of soil nutrients, such as phosphorus, potassium, and iron, furthermore, are strongly impacted by soil pH. The significant changes in bioavailable soil nutrient pools suggest further attention is needed to understand their role in sustaining plant invasions.

### Fungal community shifts due to plant invasion

It was expected that invaded soils would tend to be less diverse and support greater dominance if invasive plants stimulated the activity of specific microbes that feedback to support invader growth. Invasion, however, was associated with greater diversity and richness of fungi (and bacteria). The importance and contribution of this microbial diversity to the success of the invaders is an open question, however, and despite attempts to link microbial diversity to function, diversity in soils is large and generally difficult to interpret. It is clear, though, that certain microbial types were associated with greater abundance in invaded soil and have the potential to feedback and support the growth and reproduction of invaders. The large changes in microbial diversity, though not straightforward to interpret, require further research and consideration of how it impacts plant invader success.

Unlike the structural and functional linkages that were made associated with bacterial community change and plant invasion, fungal communities in the current study were not as clearly demarcated phylogenetically nor linked with specific processes. There were, however, very similar directional shifts in fungal community structure that help to support the findings observed for bacterial communities. Indeed, shifts in fungal community structure accounted for up to 17% of the variation in the PCoA plot (Fig 1). Fungi play critical ecosystem roles as saprotrophs, mutualists, and pathogens and though pinpointing the exact nature of the effects are not possible in the current study, the patterns of community change support the idea that plant invaders drive and are driven by a positive plant-microbial feedback model that fuel their success.

The Ascomycota showed greater abundances associated with invasion, and as the compositionally largest phylum of fungi with 64,000 species and a range of traits that include saprobe, pathogens, and mutualists, the effects of the change are likely to be functionally important [68, 69]. It is important to recognize that fungi, like bacteria, can have multiple ecological roles; for example, many mycorrhiza are also saprotrophs. Using their methodology to sort orders into an ecological context, however, Sordariales were overwhelmingly characterized as Saprobes, and the Hypocreales and Capnodiales form a mix of saprobes, plant associates and plant pathogens. So although the primary ecological changes that were observed using these methodologies are still broad, they show the potential that phylogeny has for predicting fungal ecology and the effects of plant invasion.

It is notable that a considerable amount of study has been given to the pathogenic roles played by many of the fungal taxa in our surveys. Dothideomycetes and Nectriaceae, for example, are found to play multiple antagonistic roles to plants and plant growth. It cannot be known, however, if these fungi actually play this type of role or are perhaps recruited to support plant invasion through antagonization of non-invaded plant species [70]. Whether serving as a loose plant affiliate or a plant-microbial interaction, there would be opportunity for invasive plants to disrupt plant communities if invaders themselves were less prone to the antagonistic effects of the pathogens. Research is needed to understand the nature of the changes in fungal community structure and their consequences for plant invader success.

## Conclusion

It is well known that invasive species have direct and indirect effects on the surrounding non-invaded plant community, especially through root exudates: *Centaurea* spp. [71]; *Ailanthus altissima* [72]; and *Artemisia vulgaris* [73]. Our study offers insights into microbial communities and plant invasions by showing a link between invasion and belowground community change. Functional predictions based on the phylogeny of bacteria agreed with field measurements of N turnover rates and suggest that changes in N cycling bacteria, which include nitrifiers and diazotrophs, may be a significant cog in the success of invasive plant encroachment and success into non-invaded/remnant ecosystems. If these results are further confirmed, management scenarios may soon be utilized to change the soil properties and outcome of plant-driven changes in microbial communities to help favor non-invaded plants and restore native ecosystem functions.

## Supporting Information

S1 Fig Rarefaction plots of bacterial alpha diversity for invaded and non-invaded samples using (a) chao1, (b) observed species, and (c) PD whole tree. (EPS)

S2 Fig Rarefaction plots of fungal alpha diversity for invaded and non-invaded samples using (a) chao1 and (b) observed species. (EPS)

S3 Fig KEGG pathways (level 3) predicted by PICRUSt that were significantly different between root-zone bacteria of invaded and non-invaded samples using two-sided Welch’s t-test with Storey FDR for multiple testing corrections. (EPS)

S1 Table KEGG pathways (level 2) predicted by PICRUSt that were significantly different between root-zone bacteria of invaded and non-invaded samples using two-sided Welch’s t-test with Benjamini Hochberg FDR for multiple testing corrections. I and N indicate pathway was abundant in root-zone bacteria of invaded and non-invaded samples, respectively.
(DOCX)

S2 Table KEGG pathways (level 2) predicted by PICRUSt that were significantly different between root-zone bacteria of invaded and non-invaded samples using two-sided Welch’s t-test with Storey FDR for multiple testing corrections. I and N indicate pathway was abundant in root-zone bacteria of invaded and non-invaded samples, respectively.
(DOCX)

S3 Table Top 20 abundant and significant (α< 0.05) level 3 KEGG processes by Storey FDR. First, the significant processes were descending sorted as per the average of mean relative frequency (%) in native and invasive samples. The top 20 abundant processes were categorized as belonging to native (N) or invasive (I) samples depending on the difference of mean rel. freq. (%). Finally, in each category, the processes were descending sorted as per the difference in mean rel. freq. (%) between I and N.
(DOCX)
