# Supplementary material for: Plant Invasions Associated with Change in Root-Zone Microbial Community Structure and Diversity
Source: PLoS One. 2015 Oct 27;10(10):e0141424. doi: 10.1371/journal.pone.0141424 (PMC4624766; doi:10.1371/journal.pone.0141424)
Supplement: S1 Table — I and N indicate pathway was abundant in root-zone bacteria of invaded and non-invaded samples, respectively. (DOCX) [file pone.0141424.s004.docx]

**Table S1. KEGG pathways (level 2) predicted by PICRUSt that were significantly different between root-zone bacteria of invaded and non-invaded samples using two-sided Welch’s t-test with Benjamini Hochberg FDR for multiple testing corrections.**

| Level 2 KEGG pathways (increasing order BH corrected p value) | Group |
| --- | --- |
| Energy Metabolism | I |
| Cell Motility | I |
| Amino Acid Metabolism | N |
| Immune System Diseases | N |
| Signaling Molecules and Interaction | N |
| Lipid Metabolism | N |
| Metabolism of Other Amino Acids | N |
| Transport and Catabolism | N |
| Transcription | N |

I and N indicate pathway was abundant in root-zone bacteria of invaded and non-invaded samples, respectively.
