# Supplementary material for: Plant Invasions Associated with Change in Root-Zone Microbial Community Structure and Diversity
Source: PLoS One. 2015 Oct 27;10(10):e0141424. doi: 10.1371/journal.pone.0141424 (PMC4624766; doi:10.1371/journal.pone.0141424)
Supplement: S2 Table — I and N indicate pathway was abundant in root-zone bacteria of invaded and non-invaded samples, respectively. (DOCX) [file pone.0141424.s005.docx]

**Table S2. KEGG pathways (level 2) predicted by PICRUSt that were significantly different between root-zone bacteria of invaded and non-invaded samples using two-sided Welch’s t-test with Storey FDR for multiple testing corrections.**

| Level 2 KEGG pathways (increasing order Storey corrected p value) | Group |
| --- | --- |
| Cell Motility | I |
| Cellular Processes and Signaling | I |
| Poorly Characterized | I |
| Energy Metabolism | I |
| Translation | I |
| Genetic Information Processing | I |
| Glycan Biosynthesis and Metabolism | I |
| Signal Transduction | I |
| Folding, Sorting and Degradation | I |
| Metabolic Diseases | I |
| Infectious Diseases | I |
| Signaling Molecules and Interaction | N |
| Transcription | N |
| Immune System Diseases | N |
| Biosynthesis of Other Secondary Metabolites | N |
| Amino Acid Metabolism | N |
| Transport and Catabolism | N |
| Metabolism of Other Amino Acids | N |
| Lipid Metabolism | N |
| Metabolism of Terpenoids and Polyketides | N |
| Environmental Adaptation | N |
| Digestive System | N |
| Enzyme Families | N |
| Cancers | N |
| Endocrine System | N |
| Xenobiotics Biodegradation and Metabolism | N |
| Immune System | N |

I and N indicate pathway was abundant in root-zone bacteria of invaded and non-invaded samples, respectively.
