# Supplementary material for: Plant Invasions Associated with Change in Root-Zone Microbial Community Structure and Diversity
Source: PLoS One. 2015 Oct 27;10(10):e0141424. doi: 10.1371/journal.pone.0141424 (PMC4624766; doi:10.1371/journal.pone.0141424)
Supplement: S3 Table — First, the significant processes were descending sorted as per the average of mean relative frequency (%) in native and invasive samples. The top 20 abundant processes were categorized as belonging to native (N) or invasive (I) samples depending on the difference of mean rel. freq. (%). Finally, in each category, the processes were descending sorted as per the difference in mean rel. freq. (%) between I and N. (DOCX) [file pone.0141424.s006.docx]

**Table S3. Top 20 abundant and significant (α< 0.05) level 3 KEGG processes by Storey FDR.**

| Level 3 processes significant by Storey FDR (α<0.05) | Mean rel. freq. (%) I –  Mean rel. freq. (%) N | Group |
| --- | --- | --- |
| Bacterial motility proteins | 0.195084675 | I |
| Methane metabolism | 0.148098067 | I |
| Function unknown | 0.120678949 | I |
| Secretion system | 0.103661933 | I |
| Pyruvate metabolism | 0.093091436 | I |
| Flagellar assembly | 0.07317117 | I |
| Glycolysis / Gluconeogenesis | 0.072181727 | I |
| Citrate cycle (TCA cycle) | 0.072041679 | I |
| Carbon fixation pathways in prokaryotes | 0.068991602 | I |
| Nitrogen metabolism | 0.051446033 | I |
| Protein folding and associated processing | 0.038192796 | I |
| Chromosome | 0.037170117 | I |
| Phenylalanine, tyrosine and tryptophan biosynthesis | 0.029974768 | I |
| Chaperones and folding catalysts | 0.018433534 | I |
| DNA replication proteins | 0.014744848 | I |
| Ribosome Biogenesis | 0.010685456 | I |
| Lipid biosynthesis proteins | -0.02660903 | N |
| Arginine and proline metabolism | -0.042705747 | N |
| Peptidases | -0.067498986 | N |
| Transcription factors | -0.070867248 | N |

First, the significant processes were descending sorted as per the average of mean relative frequency (%) in native and invasive samples. The top 20 abundant processes were categorized as belonging to native (N) or invasive (I) samples depending on the difference of mean rel. freq. (%). Finally, in each category, the processes were descending sorted as per the difference in mean rel. freq. (%) between I and N.
